# Water‐Saturated Ion Gel for Humidity‐Independent High Precision Epidermal Ionic Temperature Sensor

**DOI:** 10.1002/advs.202200687

**Published:** 2022-03-25

**Authors:** Hyun Woo Kim, Eunseo Kim, Joosung Oh, Hyomin Lee, Unyong Jeong

**Affiliations:** ^1^ Department of Materials Science and Engineering Pohang University of Science and Technology (POSTECH) 77 Cheongam‐Ro, Nam‐Gu, Pohang Gyeongsangbuk‐Do 37673 Republic of Korea; ^2^ Department of Chemical Engineering Pohang University of Science and Technology (POSTECH) 77 Cheongam‐Ro, Nam‐Gu, Pohang Gyeongsangbuk‐Do 37673 Republic of Korea

**Keywords:** deformation independence, humidity independence, stretchable temperature sensor, water‐saturated Ion gel, wearable sensor

## Abstract

Although ion gels are attractive sensing materials for deformable epidermal sensors or implantable devices, their sensing performances are highly affected by environmental humidity change, so that their sensing reliability cannot be secured. This study proposes a new concept of maintaining the high‐precision temperature sensing performance of highly deformable ion gel sensors. In this approach, a hydrophobic ion gel sensing layer is kept water‐saturated by attaching a hydrogel layer, rather than attempting to completely block water penetration. This study performs experimental and theoretical investigation on water concentration in the ion gel, using the analysis of mass transportation at the interface of the ion gel and the hydrogel. By using the charge relaxation time of the ionic molecules, the temperature sensor is not affected by environmental humidity in the extreme range of humidity (30%–100%). This study demonstrates a highly deformable on‐skin temperature sensor which shows the same performance either in water or dry state and while exercising with large strains (*ε* = 50%).

## Graphical abstract

A highly deformable ion gel‐based temperature sensor that is not affected by environmental humidity change is fabricated by keeping a hydrophobic ion gel sensing layer to be water saturated. The sensor shows identical high‐precision temperature sensing performance either in water or dry conditions, additionally in a stretched state when attached to the elbow.

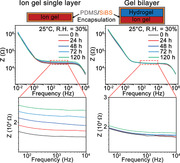

## 1 Introduction

Ion gels have attracted intensive attention as sensing materials for deformable skin‐mounted or implantable devices.^[^ ^1^, ^2^, ^3^ ^]^ The fast growing interest has been due to the unique physical characteristics (low modulus, large mechanical stretchability, and self‐healing) and the unique ionoelectric properties (controllable ionic conductivity, large electrical double layer (EDL) capacitance, and frequency‐dependent electrochemical behavior).^[^ ^2^, ^4^ ^]^ Ion gels have been used for ion conductors,^[^ ^5^, ^6^ ^]^ strain sensors,^[^ ^7^, ^8^ ^]^ touch sensors,^[^ ^9^, ^10^ ^]^ pressure sensors,^[^ ^11^, ^12^ ^]^ temperature sensors,^[^ ^13^, ^14^ ^]^ and biological chemical sensors.^[^ ^15^ ^]^ The recent concept of the ionic diode is expected to extend the possible format of ion gel‐based devices.^[^ ^16^, ^17^, ^18^ ^]^ The need for implantable devices with a low modulus comparable to the internal organs has been accelerating the research on ion gel‐based sensors.^[^ ^19^, ^20^, ^21^ ^]^ Ionovoltaic signal generation opens the possibility of soft self‐signaling sensors,^[^ ^22^ ^]^ and the organic electrochemical transistors (OECTs) are expected to allow artificial synaptic control of the transmitted potential signals.^[^ ^23^, ^24^, ^25^ ^]^

However, the moisture‐dependent ionoelectric property is a challenging drawback of the ion gel‐based sensors.^[^ ^26^, ^27^ ^]^ Small change in humidity causes a large change in the impedance profile of the ion gel, thus the sensor loses the signal reliability. This sensitive humidity dependence is mainly attributed to the change in the bulk conductivity (*σ*) and permittivity (*ε*) depending on water concentration in the ion gel.^[^ ^27^, ^28^ ^]^ The EDL capacitance change at the gel‐electrode interface can be an additional cause of the impedance profile change.^[^ ^29^, ^30^ ^]^ Encapsulation with a hydrophobic elastomer like polydimethylsiloxane (PDMS) has been investigated to minimize the moisture effect,^[^ ^31^, ^32^ ^]^ however this approach cannot completely block the moisture penetration for a long time in the current state where a perfect stretchable passivation layer has not been developed yet. A few studies have attempted to decrease evaporation‐driven weight loss of the gels. Sun et al. used an organogel with a solvent with a high boiling point and demonstrated mechanical actuation.^[^ ^33^ ^]^ They did not discuss the humidity sensitivity of the device because the dielectric elastomer actuation which is operated at a high driving voltage is relatively less affected compared to electrochemical sensors. In addition, the effect of water absorption is unavoidable in this approach. Therefore, it is a big challenge to develop a new concept making the ion gels insensitive to variations of environmental humidity.

In this study, we propose a new design concept of “water‐saturated ion gel” to remove the humidity effect on the electrical properties of ion gels, instead of attempting to completely block the moisture penetration and evaporation. We use a double layer consisting of a hydrophobic ion gel layer covered with a hydrogel layer. The hydrophobic ion gel layer can maintain its maximum water solubility while environmental humidity changes, thus its electrical properties are not affected in a wide range of relative humidity (R.H. = 30%–100%) which corresponds to real‐life humidity experience. The maintenance of water concentration in the ion gel layer is investigated by experimental and computational methods by analyzing mass transportation of water at the interface of the gel bilayer. We demonstrate a stretchable skin‐mountable temperature sensor that is independent of external humidity and external mechanical forces.

### 1.1 Fabrication of the Hydrogel/Ion Gel Bilayer Sensor

**Figure 1:**
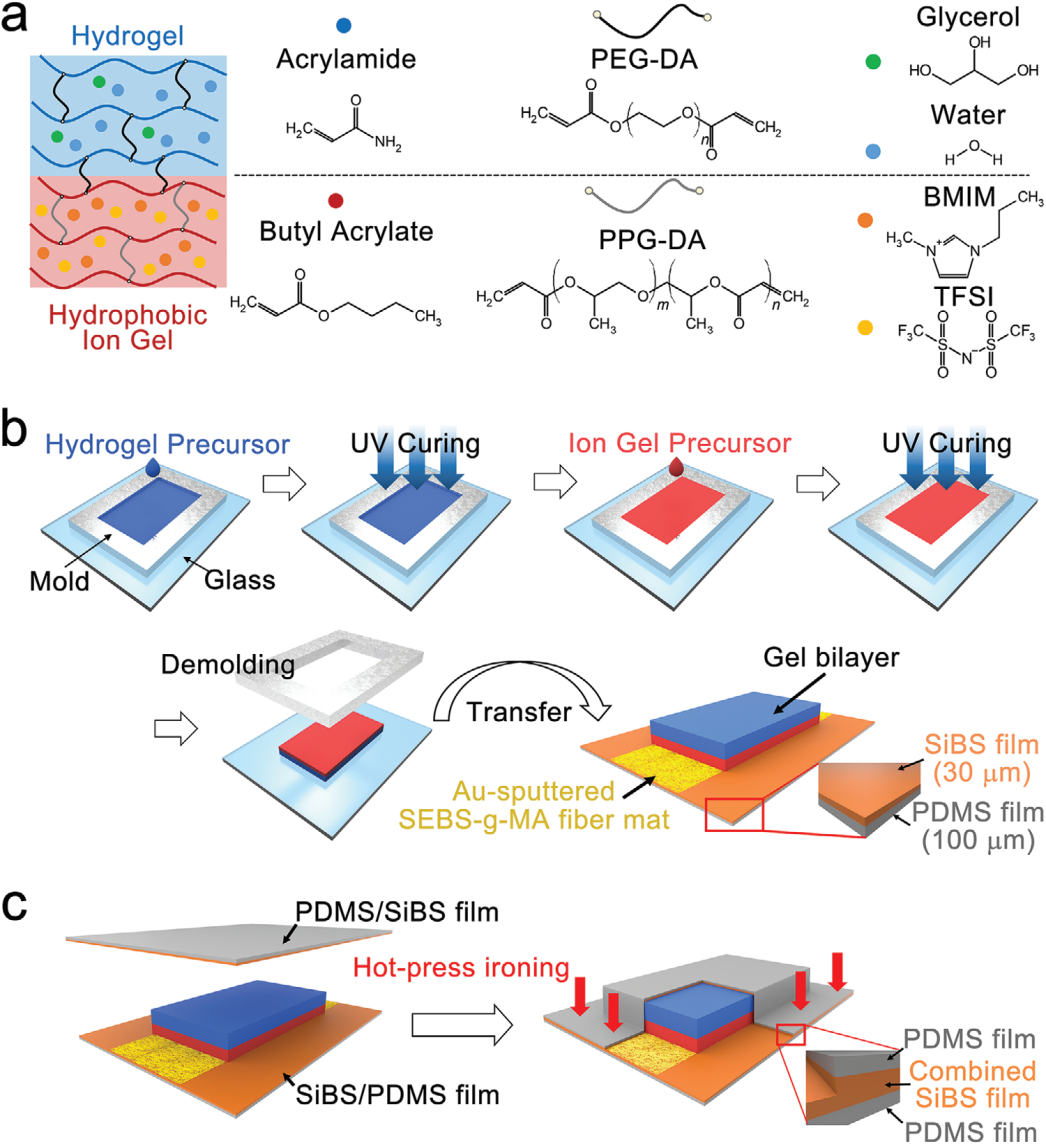
a) Scheme of the gel bilayer consisting of a hydrophobic ion gel layer (bottom) and a hydrogel layer (top). The two layers are chemically bound. The molecules are constituents in each layer. b) The process to fabricate the gel bilayer on a pair of coplanar stretchable electrodes. c) Encapsulation process to cover the gel bilayer with a stretchable hydrophobic double layer film consisting of a PDMS film and an SiBS film. The double layer films cover the gel bilayer and combined with the viscoplastic SiBS layer through hot pressing.

Instead of trying to completely passivate the ion gel, we kept a hydrophobic ion gel saturated with water by covering a hydrogel layer on the ion gel (**Figure** **1**a). A hydrogel precursor solution was prepared by mixing acrylamide (Aam), poly(ethylene glycol) diacrylate (PEG‐DA), 2‐hydroxy‐2‐methylpropiophenone (HMPP), and an aqueous glycerol solution (8 wt%). Glycerol was added to form the hydrogen bonding with water and the acrylamide so that the water retention of the hydrogel could be enhanced.^[^ ^34^ ^]^ An ion gel precursor solution was composed of butyl acrylate (BA), poly(propylene glycol) diacrylate (PPG‐DA), HMPP, and 1‐buyl‐3‐methylimidazolium bis(trifluoromethylsulfonyl)imide (BMIM:TFSI). Because the water solubility of BMIM:TFSI is about 1.3 wt%, the ion gel is hydrophobic and can be easily saturated with water.^[^ ^35^ ^]^ Figures 1b,c illustrate the sensor fabrication including the hydrogel/ion gel bilayer. The hydrogel precursor solution was introduced in a stainless‐steel mold, then UV was irradiated to partially form the hydrogel. The ion gel precursor solution was introduced on the hydrogel layer in the mold, and then UV was irradiated. The hydrogel layer was crosslinked together with the ion gel layer during the second UV curing. The diacrylate (DA) from PEG‐DA and PPG‐DA played as the crosslinking agent at the interface, hence the two layers had chemically bound for enough adhesion. A peeling test was performed to check the adhesion between the gel layers (Figure S1, Supporting Information). The peeling test result between the commercial 3M 1533 tape and a slide glass is compared as a reference. In order to check whether the possible mutual diffusion of water and ionic molecules affects the interfacial stability, the peeling tests were performed immediately after the fabrication of the bilayer gel and after 24 h. The peeling test results of the gel bilayer were comparable to the 3M tape test. There was no notable difference between the two samples and we could not find any precipitation observed by optical microscope, which indicates the possible diffusion of ionic molecules and water is not considered to affect the interfacial stability.

We prepared a double layer stretchable substrate (SiBS/PDMS), by spin‐coating polystyrene‐block‐poly(isobutylene)‐block‐polystyrene (SiBS) (30 µm in thickness) on a PDMS film (100 µm in thickness). An electrospun nanofiber mat made of polystyrene‐block‐poly(ethylene‐ran‐butylene)‐block‐polystyrene grafted with maleic anhydride (SEBS‐g‐MA) was placed on the SiBS/PDMS substrate. Au was sputtered on the SEBS‐g‐MA nanofiber mat through a patterned mask to form a pair of stretchable electrodes. The stainless‐steel mold for the gel bilayer was removed and the gel bilayer was transferred onto the Au electrodes. The gel bilayer was covered with another double layer substrate (PDMS/SiBS), then sealed by ironing at 140 °C (Figure 1c). Owing to the thermoplasticity of the SiBS layer, the cover film and the bottom substrate were strongly bonded and sealed the gel bilayer. It is notable that SiBS was chosen as the cover layer because of its low water permeability.^[^ ^32^, ^36^ ^]^

### 1.2 Water Concentration in the Ion Gel Layer of the Gel Bilayer

**Figure 2:**
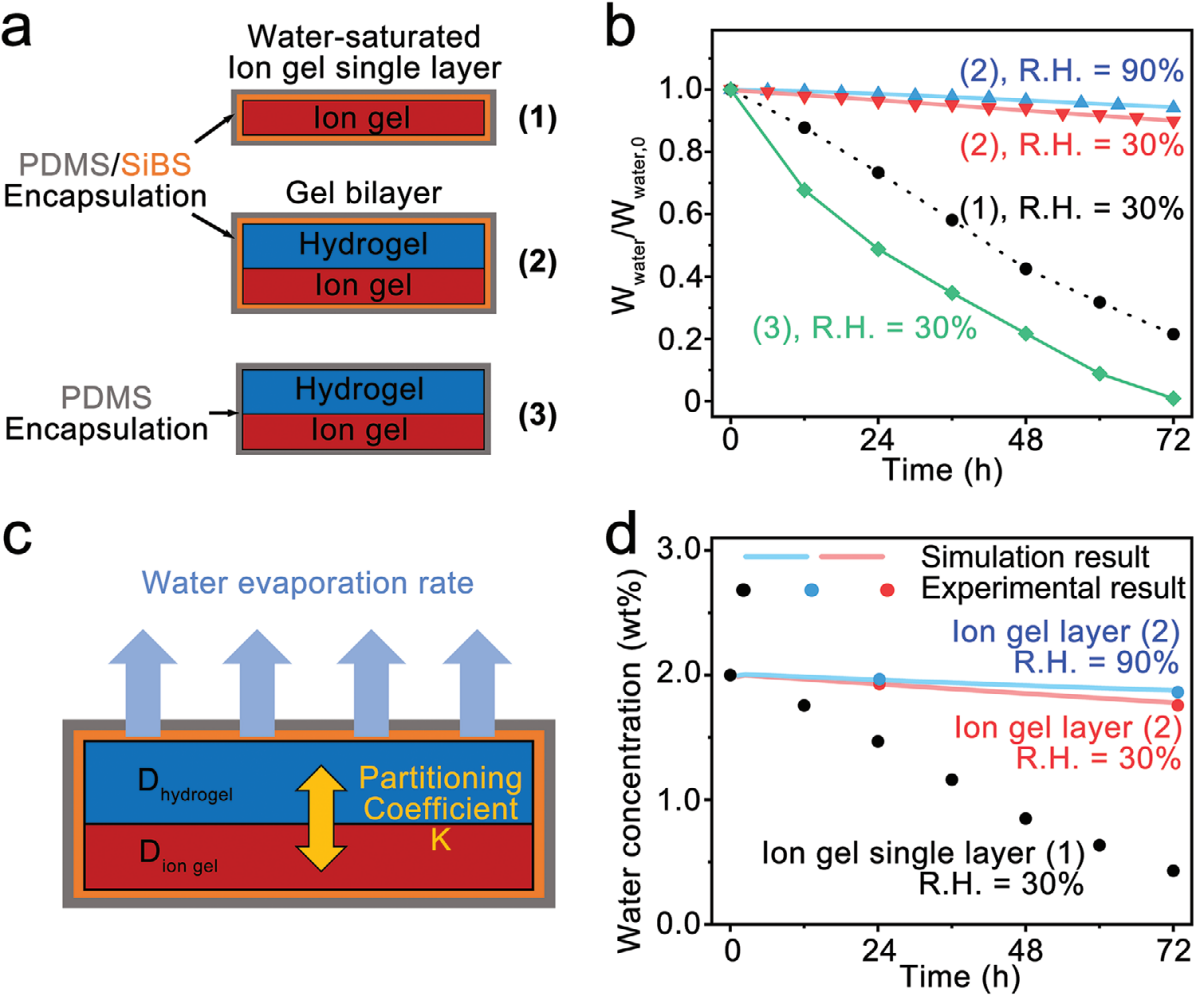
a) Scheme of the sample design for testing water evaporation. The encapsulation was either a PDMS (100 µm)/SiBS (30 µm) double layer (1,2) or a PDMS (100 µm) single layer (3). For the gel, the ion gel single layer or the gel bilayer was tested. b) Relative weight of water; in the gel bilayer (2) with the PDMS/SiBS encapsulation at R.H. = 30% (red) and R. H. = 90% (blue), in the gel bilayer (3) with the PDMS encapsulation (green), and in the ion gel single layer (1) with the PDMS/SiBS encapsulation (black). c) Scheme of the simulation parameters to calculate the water concentration in the ion gel and hydrogel layer. d) Computational (solid line) and experimental (symbols) results of the water concentration change in the ion gel layer of the gel bilayer at R. H. = 30% (red) and 90% (blue). The water concentration change in the ion gel single layer with the PDMS/SiBS encapsulation at R.H. = 30% is also shown for comparison.

Humidity change leads to outward/inward flux of water molecules in the top hydrogel layer, which in turn may cause concentration change of water in the bottom ion gel layer. Maintaining a constant water concentration in the ion gel is the key to achieve the humidity‐independent sensor, which is enabled by the addition of a hydrogel layer atop of the ion gel. To verify this hypothesis, we investigated the water evaporation rate of the single ion gel layer and the gel bilayer which had the PDMS (100 µm)/SiBS (30 µm) or the PDMS (100 µm in thickness) encapsulation layer (**Figure** **2**a). The conditions of the gel layer and encapsulation layer described in the figure are numbered as (1)–(3). We measured the initial weight of the gels and the weight change of the encapsulated systems to monitor the relative change of water in the gels at 25 °C for 72 h under the dry (R.H. = 30%) and humid (R.H. = 90%) conditions (Figure 2b). We observed an 80% loss of water from the water‐saturated ion gel single layer with the PDMS/SiBS encapsulation (1) after 72 h in the dry condition. Comparatively, 10% of water loss was observed from the gel bilayer with the same PDMS/SiBS encapsulation (2) in the dry condition, and only 5% loss was measured in the humid condition. It is notable that the PDMS passivation (3) caused complete loss of water in the gel bilayer within 72 h in the dry condition.

For theoretical modeling of water concentration in the gel layer, the diluted species module in COMSOL software was used. We assumed uniform initial water concentration, constant diffusion coefficient, and constant temperature. We also assumed one‐dimensional diffusion process only in the thickness direction, following Fick's second law, $\frac{\partial C}{\partial t}=\;D\nabla^{2}C$, where *C* and *D* are the water concentration and the diffusion coefficient of water in the gel. The dimension of the ion gel layer was 770 µm in thickness and 3 cm × 1 cm in a plane, hence the side wall of the ion gel was negligible in the total surface area. In addition, the four sides of the gel bilayer were blocked by the extended film of the encapsulating cover layer, hence water evaporation from the side walls of the ion gel layer was not considered.

The temporal evaporation rate was measured by differentiating the experimental results on the weight change with time. The time‐dependent evaporation flux, *f*(*t*), was used in the flux boundary condition at the top surface of the hydrogel layer.

(1) $$\left(-D_{hydrogel}\nabla C_{w,\mathrm{hydroge}l}\right)\cdot n=\;\;f\left(t\right)\;\mathrm{at}\;\partial \;\Omega {\;}^{\mathrm{hydrogel}/\mathrm{air}}$$

The stiff spring model was used at the hydrogel‐ion gel interface ∂Ω^hydrogel,ion gel ^ to acquire the continuous water flux that is described as following Equations (2) and (3),

(2) $$\begin{array}{ccc} & & \left(-D_{hydrogel}\nabla C_{w,\mathrm{hydrogel}}\right)\cdot n\;=\;M\left(C_{w,\;\mathrm{ion}\;gel}-KC_{w,\mathrm{hydrogel}}\right)\\ & & \;\;\;\;\;\;\mathrm{at}\;\partial \;\Omega^{\;\mathrm{hydrogel}/\mathrm{ion}\mathrm{gel}} \end{array}$$

(3) $$\begin{array}{ccc} & & \left(-D_{ion\;gel}\nabla C_{w,\mathrm{ion}\;gel}\right)\cdot n\;=\;M\left(KC_{w,\mathrm{hydrogel}}-C_{w,\mathrm{ion}\;gel}\right)\\ & & \;\;\;\;\;\;\mathrm{at}\;\partial \;\Omega^{\;\mathrm{ion}\mathrm{gel}/\mathrm{hydrogel}} \end{array}$$

where **n** is the normal vector, and *M* is a sufficiently large non‐physical number. We implemented the equilibrium partitioning coefficient, $K\;=\;\frac{C_{w,\text{ion}\;\text{gel}}}{C_{w,\;\mathrm{hydrogel}}}$, to account for the fraction of water joining in each phase of the gel bilayer. Water is distributed to achieve thermodynamic equilibrium at the interface. Since the partitioning coefficient is the ratio of the water concentration in the ion gel and the hydrogel, the ratio of the partial chemical potentials of water molecules in the ion gel layer (a very low water concentration regime) and the hydrogel layer (a high water concentration regime) is regarded to be same with the partitioning coefficient, hence understanding the water migration behavior is possible with the partitioning coefficient.

The model described above requires diffusion coefficients of water, partitioning coefficient, and evaporation rate through the passivation layer (Figure 2c). We acquired the diffusion coefficients of water in each gel medium from diffusion‐ordered spectroscopy (DOSY) which is a well‐established nuclear magnetic resonance (NMR) methodology selectively measuring the diffusion coefficient of the target species.^[^ ^37^, ^38^ ^]^ Figure S2, Supporting Information, compares the ^1^H NMR spectra of a dry ion gel and the water‐saturated ion gel with the NMR DOSY and shows the ^1^H NMR with DOSY of the hydrogel. The diffusion coefficients of water in the ion gel and hydrogel were 2.53 × 10^−4^ mm^2^ s^−1^ and 1.25 × 10^−3^ mm^2^ s^−1^, respectively. The measurement of the water diffusion coefficients is detailed in Text S1, Supporting information.

We determined the time‐dependent evaporation rate in the gel bilayer with PDMS/SiBS passivation layer (2) under the dry and humid conditions by differentiating the polynomial fitting of the relative water loss data. The evaporation rate in the dry condition decreased from 0.51  to 0.46 mg h^−1^ after 72 h, and it decreased from 0.31  to 0.25 mg h^−1^ in the humid condition. The equilibrium partitioning coefficient (*K*) of 0.025 at 25 °C was obtained by measuring the water concentration of each layer after removing the PDMS/SiBS encapsulation (Figure S3, Supporting information). Comparatively, the water concentration in the ion gel single layer (1) decreased rapidly down to 0.4% after 72 h. The simulated temporal changes of water concentration in the ion gel layer (solid lines) were in excellent agreement with the measured values (symbols) (Figure 2d), showing a negligible change in both the dry and humid conditions. The data for 5 days are shown in Figure S4a, Supporting information. On the basis of the results, the possible time period the hydrogel layer can supply water to the ion gel in a continuous desert condition (R.H. = 30%) can be estimated to be longer than 3 days. Water concentration in the ion gel was proportional to the water composition in the hydrogel, hence decreasing as water in the hydrogel continued to evaporate (Figure S4b, Supporting information). Since water evaporation in the hydrogel for consecutive 5 days in the dry condition did not result in significant impedance change in the ion gel, the sensor is expected to be used in daily life without worry about unwanted signal change. Once the sensor was completely dried, it took about 30 days in the dry condition, the sensor was bent due to the volume change difference and could not restore the initial shape even in a humid environment, hence reuse of the sensor after complete drying was not possible. Overall, the results confirm that the presence of the hydrogel layer plays an important role in maintaining the water concentration of the ion gel.

### 1.3 Impedance Profile of the Water Saturated Ion Gel

**Figure 3:**
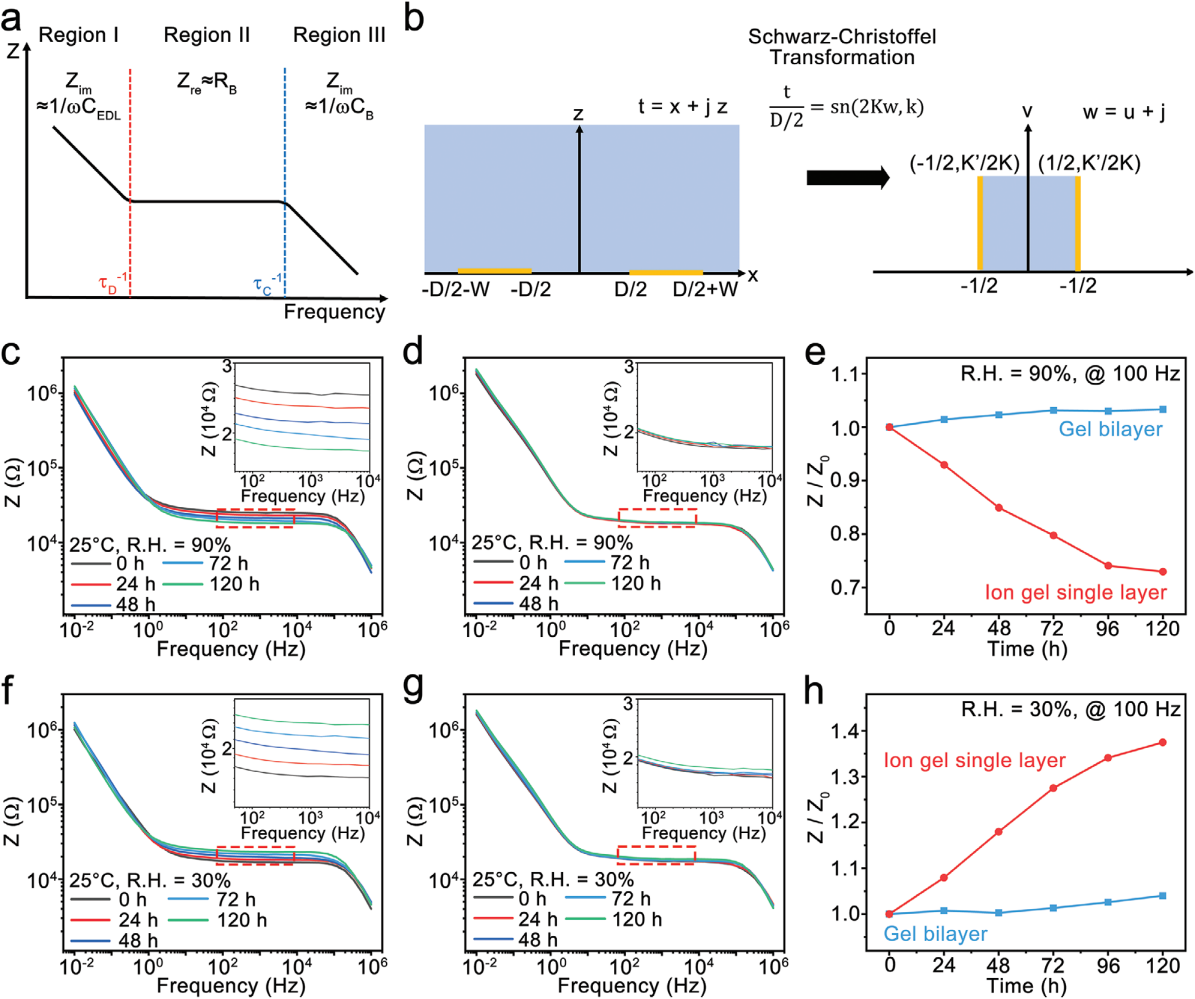
a) Scheme of the Bode plot of the ion gel which is dependent on measurement frequency. b) Scheme of the Schwarz‐Christoffel transformation for the coplanar electrode structure. c,d) Bode plot of the ion gel single layer (c) and the gel bilayer (d) in a hummid condition (R.H. = 90%). e) Relative impedance change (*Z*/*Z*_0_) of the ion gel single layer (red) and the gel bilayer (blue) at the humid condition. (f,g) Bode plot of the ion gel single layer f) and the gel bilayer g) in a dry condition (R.H. = 30%). h) Relative impedance change (*Z*/*Z*_0_) of the ion gel single layer (red) and the gel bilayer (blue) at the dry condition. The environmental temperature was 25 °C.

The impedance (Z) profile of a non‐Faradaic ion gel system shows a strong dependence on measurement frequency (**Figure** **3**a). The frequency dependence is largely dominated by EDL in the low‐frequency Region (I), by the ion migration in the middle frequency Region (II), and by the ion or dipole polarization in the high‐frequency Region (III).^[^ ^39^, ^40^ ^]^ The transition frequency between Region I and Region II (I → II) presents the dielectric relaxation, and the other transition frequency between region II and region III (II → III) indicates the charge relaxation. These behaviors can be interpreted with the equivalent circuit model that is composed of CPE by EDL (CPE_EDL_), bulk resistance (*R*_B_) by ion migration, bulk capacitance (*C*_B_) by ion polarization, and electrode resistance (*R*_E_) (Figure S4, Supporting information). *R*_B_ and *C*_B_ can be obtained from the approximate relationships, $C_{\mathrm{Bulk}}\;\approx \;\frac{1}{\omega Z_{III,im}}\;$and *R*_Bulk_ ≈  *Z*_*II*,*re*_.^[^ ^13^ ^]^ Ion gel‐based sensors have mostly used the top‐bottom parallel electrode setup, in which *R*_B_ and *C*_B_ can be obtained by $R_{B}=\;\rho \;\frac{d}{A}\;$and $C_{B}=\;\varepsilon \;\frac{A}{d}$. Previously, we verified that high‐precision real‐time temperature sensing is possible by using the ion gel as a sensing material in the top‐bottom electrode setup.^[^ ^13^ ^]^ We utilized the change of the charge relaxation time (*τ*) by temperature change. We also demonstrated that *R*_B_ can be measured at *ω*_II_ in the Region II and *C*_B_ can be calculated at *ω*_III_ in the Region III, then *τ* can be obtained by multiplying R_B_ and C_B_, $\tau \;=\;R_{B}\times \;C_{B}=\;\rho \;\frac{d}{A}\times \varepsilon \;\frac{A}{d}=\;\rho \cdot \varepsilon$.

Because *τ* is an intrinsic variable that is only dependent on the changes of the permittivity (*ε*) and the resistivity (*ρ*) of the bulk ion gel, thus it is not affected by dimensional changes.

In this study, we used the coplanar electrode setup for structural simplicity (Figure 3b). *R*_B_ and *C*_B_ in the coplanar structure can be calculated by using the Schwarz‐Christoffel transformation, $R_{B}=\;\rho \;\frac{2K(k)}{K(k^{\prime })\;L},$ $C_{B}=\;\varepsilon \frac{K(k^{\prime })\;L}{2K(k)}$, where *L* is the length of the electrode, *K*(*k*) is the complete elliptic integral of the first kind, *k* is the geometry‐dependent factor, and *k*’ is the complementary geometry‐dependent factor.^[^ ^41^ ^]^ Through the Schwarz‐Christoffel transformation, the dimensional variables in the coplanar electrode structure can be converted to the dimensional variables in the rectangle‐shaped parallel electrode structure with the geometry‐dependent factor *k* (≡ *D*/(*D* + 2*W*)) and the elliptic integral *K*(*k*). *τ* can be also obtained from the transformation by $\;\tau =\;R_{B}\times \;C_{B}=\;\rho \;\frac{2K(k)}{K(k^{\prime })\;W}\times \varepsilon \;\frac{K(k^{\prime })\;W}{2K(k)}=\;\rho \cdot \varepsilon$, indicating that *τ* is defined exactly in the same way as the parallel electrode setup, thus not affected by dimensional changes. Detailed explanation about the Schwarz‐Christoffel transformation is provided in Text S2, Supporting Information.

Figures 3c,d exhibit the electrochemical impedance spectroscopy (EIS) profiles for the ion gel single layer (c) and the hydrogel/ion gel bilayer (d) when measured at a relative humidity (R.H.) of 90% and room temperature for 120 h. The impedance profile of the ion gel single layer shifted down in Region II. We obtained the similar down‐shift in the humid condition when other ion gels were used, including lithium bis(trifluoromethylsulfonyl)imide (Li:TFSI), 1‐ethyl‐3‐methylimidazolium bis(trifluoromethylsulfonyl)imide (EMIM:TFSI), BMIM:TFSI, 1‐butyl‐3‐methylimidazolium tetrafluoroborate (BMIM:BF_4_) (Figure S5, Supporting Information). On the other hand, the profile of the gel bilayer showed negligible changes. Figure 3e compares the change of the relative impedance (*Z*/*Z*_0_) of the ion gel single layer and the gel bilayer at 100 Hz. The relative impedance of the single layer ion gel rapidly decreased to 0.7 in 120 h, meanwhile, the relative impedance of the gel bilayer showed a marginal increase (1.03) in 120 h. Immediately after the measurements at R.H. = 90% for 120 h, the samples were tested in a dry condition (R.H. = 30%) for another 120 h. Figures 3f,g exhibit the changes of the EIS profile of the ion gel single layer (f) and the gel bilayer (g) in the dry condition. The impedance profile of the ion gel single layer shifted up in Region II, whereas the profile of the gel bilayer maintained the same during 120 h. The relative impedance (at 100 Hz) of the ion gel single layer sharply increased to 1.4 (Figure 3h), however, the relative impedance of the gel bilayer showed a slight increase to 1.04 for 120 h. The average R.H. in human life varies in the range of 30%–100%. Since the average R.H. of the desert climate region such as Las Vegas is about 30%,^[^ ^42^ ^]^ the consecutive test for 120 h at R.H. = 30% is an extreme dry condition. On the basis of the results, the ion gel single layer is sensitive to humidity change even with a good passivation layer, however, the hydrogel/ion gel bilayer is independent of the external humidity change.

### 1.4 Humidity‐Independent and Deformation‐Independent Temperature Sensor

**Figure 4:**
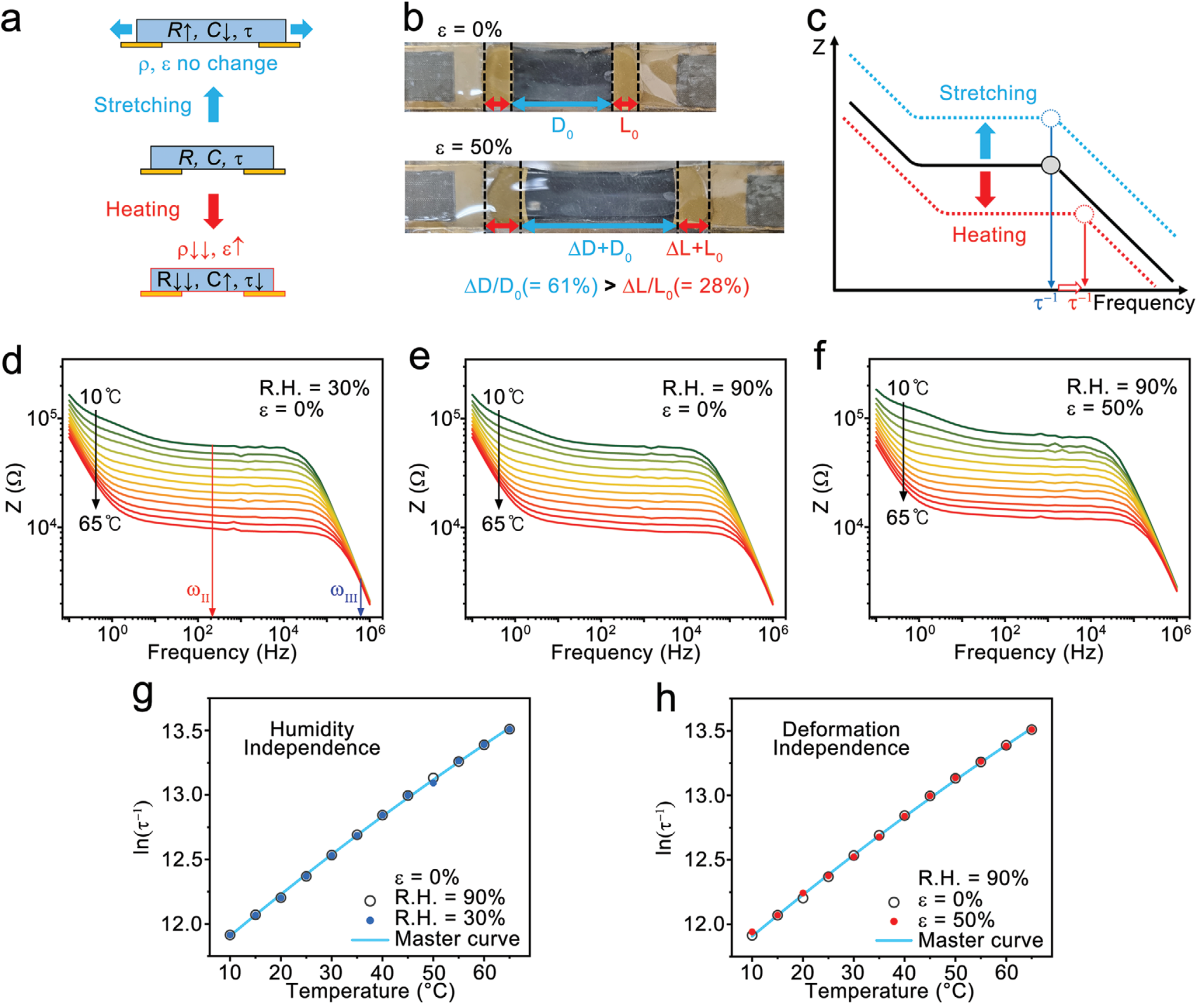
a) Scheme showing the changes of electrical properties in the ion gel under stretching and heating. The resistivity (*ρ*) and permittivity (*ε*) do not change under stretching. b) Images of the ion gel sensor before and after uniaxial stretching to *ε* = 50%. c) Scheme of the Bode plot showing a parallel upshift of the impedance profile by stretching and a non‐parallel down‐right shift by heating. d–f) Impedance profiles of the ion gel sensor according to temperatures (10–65 °C) without stretching (*ε* = 0%) in the dry condition (R.H. = 30%) (d) and humid condition (R. H. = 90%) e), and under stretching and humid condition (*ε* = 50%, R.H. = 90%) (f). g) Changes of ln(*τ* ^−1^) at various temperatures (10–65 °C) at the dry and humid conditions without stretching. The results show the humidity independence of the sensor. h) Changes of ln(*τ* ^−1^) at various temperatures (10–65 °C) at the humid condition with and without stretching. The results indicate the deformation independence of the sensor. The cyan lines are the master curves fitting the data (ln(*τ* ^−1^) = 11.5733 + 0.034 *T* – 0.0000632 *T* ^2^). *R* ^2^ value from the 12 points was 0. 9996.

When the ion gel sensor is uniaxially stretched, the resistance of ion gel increases and capacitance decreases due to change of geometrical factors, however, the resistivity (*ρ*) and permittivity (*ε*) do not change (**Figure** **4**a). In the case of 50% stretched ion gel sensor, the relative elongation between electrodes (ΔD/D_0_) was 61%, and the relative elongation of the electrodes (Δ*L*/*L*_0_) was 28% (Figure 4b). The relatively large elongation of the gel between the electrodes is attributed to the greater force required for elongation due to the increased thickness by attaching Au‐sputtered SEBS‐g‐MA nanofiber electrodes (Figure S6, Supporting Information). Since the impedance profile is mainly affected by the elongation of the gel bilayer, mechanical stretching moves the impedance profile upward, however, it does not change the transition frequency (*τ* ^−1^) (Figure 4c). On the contrary, temperature increase enhances the kinetic energy of the ions, thus the impedance profile moves downward and simultaneously *τ* ^−1^ shift to the right.

Figures 4d,e exhibit the temperature dependence of the EIS profiles from 10 to 65 °C in a dry condition (R.H. = 30%) (d) and in a humid condition (R.H. = 90%) (e). Mechanical stretching was not applied in both cases (*ε* = 0%). The sensor showed almost identical EIS profiles in both humidity conditions. *R*_B_ and *C*_B_ were measured at two frequencies (*ω*_II_ = 316 Hz, *ω*_III_ = 621 300 Hz). From the measured *R*_B_ and *C*_B_ at each temperature, we obtained the transition frequency (*τ* ^−1^) in the range of 10°C – 65 °C and created a master curve for the relationship between the transition frequency, ln(*τ* ^−1^) and temperature, *T* with a polynomial fitting. From the master curve, the average error of the temperature measurement between humid and dry conditions was 0.12 °C. The master curve was applicable to both the dry and humid conditions, indicating the sensor could work properly without affected by external humidity conditions (Figure 4g). The ion gel temperature sensor could be stretched up to *ε* = 50%. Figure 4f exhibits the EIS profiles when the temperature was varied in the range of 10 and 65 °C at the stretched state (*ε* = 50%) and the humid condition (R.H. = 90%). Although EIS profiles changed when the sensor was stretched, *τ* ^−1^ maintained the same with the value at *ε* = 0%. Figure 4h shows a master curve excellently applicable to both the stretched and non‐stretched states, indicating the sensor is independent of the external stretching.

**Table** **1** compares the temperature sensing performance with the reported deformable temperature sensors; temperature coefficient (TC), error under stretching, and humidity effect. The measurement sensitivity of our sensor (TC = 1.5% per °C) is relatively high and the maximum stretchability of this work is outstanding compared to the reported values of stretchable temperature sensors. Despite the high uniaxial strain (*ε* = 50%), the average error (0.13 °C) was smallest than the other sensors. The reported stretch sensors were used under one humidity condition and either did not report measurement errors due to humidity changes or were significantly affected by humidity. Some sensors showed very small influence by the humidity change, but they were not deformable. The average measurement error (0.12 °C) in a large humidity range (R.H. = 30%–100%) enables accurate temperature measurement when the sensor was put on the skin, which is a considerable advance in the deformable temperature sensor.

**Table 1:** Comparison of the temperature coefficient (TC), error under stretching, humidity sensitivity of the deformable temperature sensors

Material (sensing type)	TC [ per °C]	Error under stretching	Humidity effect (Humidity change)	Ref.
Ionic liquid (conductance)	3.90%	3.3 °C (*ε* = 30%)	1.6 °C (13–87%)	[43]
R‐GO, Polyurethane (resistance)	1.34%	2.24 °C (*ε* = 5%)	–	[44]
Au (Resistance)	0.34%	1 °C (*ε* = 15%)	–	[45]
Graphene oxide (resistance)	0.80%	0.37 °C (*ε* = 50%)	–	[46]
Mg (Resistance)	0.25%	≈0.2% (*ε* = 10%)	–	[47]
Graphene‐nanocellulose (resistance)	1.05%	≈30 °C (*ε* = 10%)	–	[48]
Au/Cr (resistance)	0.28%	≈3 °C (*ε* = 3%)	–	[49]
Polycarbonate (current)	5.50%	Rigid	0 °C (Immersed in the water)	[50]
Cellulose‐PPy (capacitance)	3.70%	Flexible	17.5 °C (5–45%)	[51]
PEDOT:PSS (resistance)	0.77%	Flexible	≈1 °C (30–80%)	[52]
Ion gel (charge relaxation time)	10.40%	0.29 °C (*ε* = 50%)	–	[13]
Ion gel (charge relaxation time)	1.50%	0.13 °C (*ε* = 50%)	0.12 °C (30–90%)	This work

**Figure 5:**
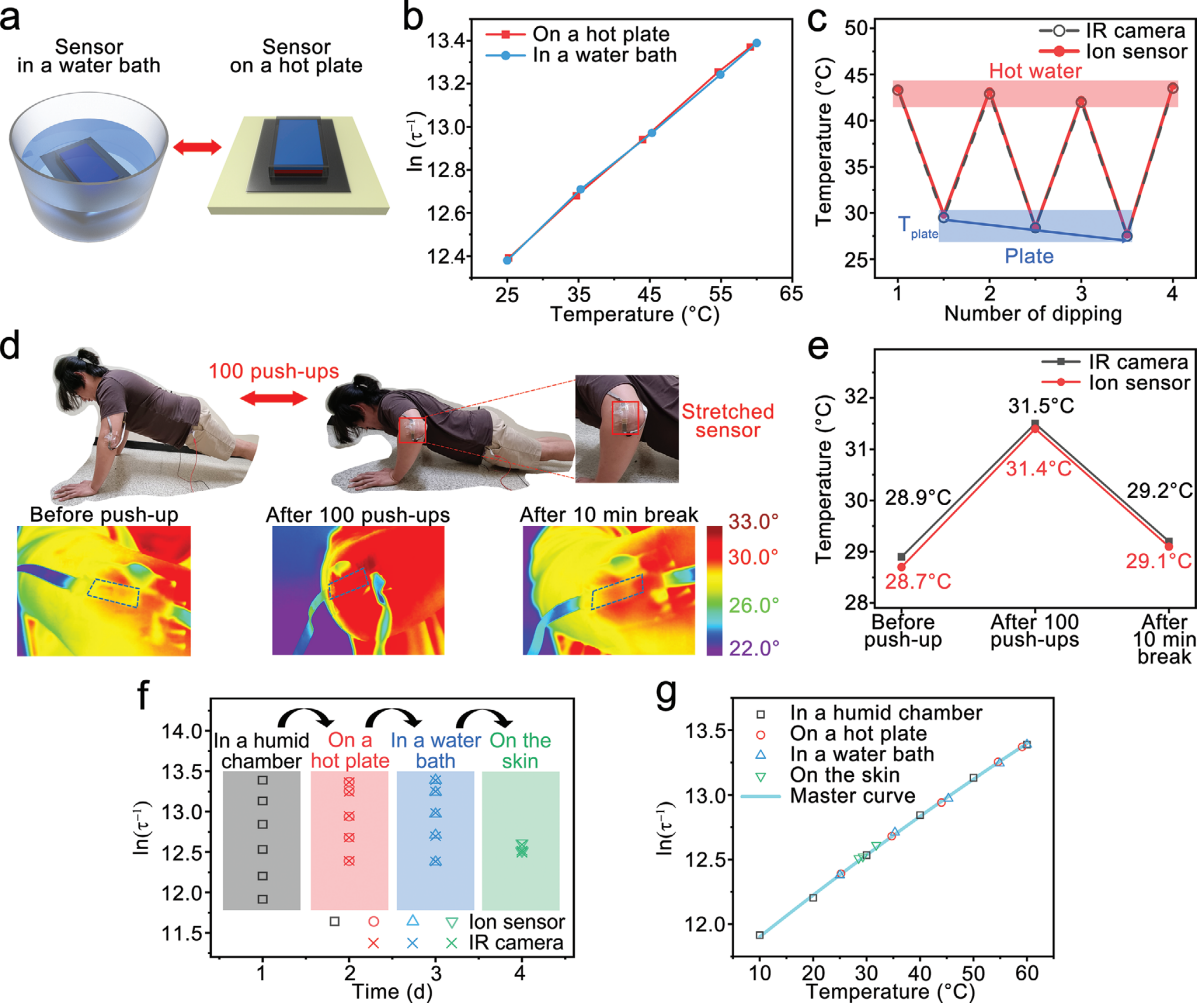
a) Scheme showing the cyclic test of soaking in a water pot and drying on a hot plate. b) Changes of ln(*τ* ^−1^) with respect to the temperature of the water pot and the hot plate. c) Measured temperatures with the ion gel sensor (red solid dot) and the IR sensor (black empty dot) during the soaking‐and‐drying cycles. The gradual decrease of the hot plate temperature was due to the air cooling. d) Camera images of 100 push‐ups while attaching the ion gel sensor to the elbow. IR thermal images were taken before push‐up, after 100 push‐ups, and after 10 min break. e) Temperature changes were measured with the ion gel sensor and IR camera at the three exercise states. f) Temperature measurement by the ion gel sensor for 4 days in various conditions (in a chamber, on a hot plate, in a water bath, and on the skin) each day. The measured charge relaxation times are hollow symbols and the calculated charge relaxation times using the master curve from the IR temperature acamera are marked by ‘ × ’. g) The measured charge relaxation time by the ion gel sensor and their converted temperatures in the various conditions. The solid line is the master curve.

The humidity independence of the sensor was demonstrated by measuring temperature in two environments (dipped in a water bath, placed on a hot plate) (**Figure** **5**a). The temperature of the water and hot plate was varied from 25 to 60 °C. The charge relaxation frequencies (*τ* ^−1^) measured in both environments were the same at each measured temperature (Figure 5b). When we compare the temperatures measured with our ion‐gel sensor and IR sensor while repeating the measurement in the water bath and on the hot plate, both measured temperatures were identical (Figure 5c). Since the ion gel sensor was deformation‐independent, it was used to measure body temperature while exercising (100 times push‐up) (Figure 5d). The sensor was attached to the elbow with double‐sided medical tape (3M 9834). The measured temperatures with the ion‐gel sensor and the IR sensor showed only a small difference (<0.2 °C) before and after exercise regardless of whether the ion gel sensor was stretched or not during exercise (Figure 5e). The stretched sensor had a higher resistance and lower capacitance compared to the unstretched sensor at the same temperature, however, the charge relaxation time was kept constant regardless of the deformation (Figure S7, Supporting Information). Because the bilayer ion gel exhibited long‐term humidity‐independent stability for 5 days as shown in Figure 3, the temperature sensor also exhibited long‐term (4 days) stable performance when exposed to various conditions; consecutively in a humid chamber, on a hot plate, in a water bath, and on the skin on each day (Figure 5f). The charge relaxation times (hollow symbols) were measured in the different conditions during 4 days. The charge relaxation times (×) corresponding to the temperature measured by the IR camera were also calculated on the basis of the previous master curve result. The measured relaxation times were in excellent agreement with the calculated values by IR. The charge relaxation time and its converted temperature obtained in the different conditions during 4 days fell into the master curve with negligible error (Figure 5g), indicating the long‐term stability of the sensor in various environments.

## 2 Conclusion

We propose an ion gel‐based skin‐mounted stretchable temperature sensor that is independent of environmental humidity change and geometrical deformation caused by an external force. The hydrogel/ion gel bilayer maintained the ion gel layer saturated with water in both the dry and wet conditions, thus not affected by the environmental humidity change. The constant water concentration in the ion gel layer was confirmed by computational calculation and experimental results. Since the variable for the temperature sensing was the charge relaxation frequency (*τ* ^−1^) which is an intrinsic variable, the sensor performance was not affected by mechanical deformation. The temperature sensor can provide precise body temperature without being affected by humidity change and body motions.

## 3 Experimental Section

### Fabrication of the Gel Bilayer

Acrylamide (Aam) (> 99%, Sigma Aldrich), poly(ethylene glycol) diacrylate (PEG‐DA) (Sigma Aldrich), 2‐hydroxy‐2‐methylpropiophenone (HMPP) (Sigma Aldrich), and an aqueous glycerol solution (8 wt%) (Sigma Aldrich) were mixed to prepare hydrogel precursor solution in a weight ratio of 150:3:4:1150. An ion gel precursor solution was prepared by mixing butyl acrylate (BA) (Sigma Aldrich), poly(propylene glycol) diacrylate (PPG‐DA) (M_n_ = 800, Sigma Aldrich), HMPP (Sigma Aldrich), and 1‐butyl‐3‐methylimidazolium bis(trifluoromethylsulfonyl)imide (BMIM:TFSI) (>98%, Sigma Aldrich) in a weight ratio of 60:1:1:40. The hydrogel precursor solution (440 µL) was poured in a stainless‐steel mold (10 mm x 30 mm x 2 mm) and UV irradiation (72.8 mW, 365 nm) was applied for 100 s. The ion gel precursor solution (280 µL) was poured on the hydrogel layer in the mold. UV was irradiated for 120 s on the ion gel precursor.

### Fabrication of the Water‐Saturated Ion Gel Single Layer

A water‐saturated ion gel precursor solution was prepared by mixing the ion gel precursor (the same with the ion gel precursor for the gel bilayer) and deionized water in a weight ratio of 98:2. The mixture was vortexed for 5 min to secure fine dispersion of water in the ion gel precursor. Immediately after mixing, the water‐saturated ion gel precursor solution (720 µL) was poured in a stainless‐steel mold (10 mm × 30 mm × 2 mm) and UV irradiation (72.8 mW, 365 nm) was applied for 120 s.

### Encapsulation of the Gel Bilayer

Polystyrene‐block‐poly(isobutylene)‐block‐polystyrene (SiBS) (SIBSTAR 103T, Kaneka, Japan) was dissolved in toluene (Samchun, Korea) (15 wt% solution). PDMS (10:1, SYLGARD 184, Dow chemical) was spin‐coated and thermally cured at 130 °C for 1.5 h. SiBS solution was spin‐coated on a PDMS film (100 µm). The thickness of the SiBS film was 30 µm. An electrospun nanofiber mat (100 µm in thickness) made of polystyrene‐block‐poly(ethylene‐ran‐butylene)‐block‐polystyrene grafted with maleic anhydride (SEBS‐g‐MA, Sigma Aldrich) was covered on the SiBS/PDMS substrate and heat‐treated on the hot plate (80 °C, 2 h). Au was sputtered (20 mW, 400 s) on the SEBS‐g‐MA nanofiber mat through a patterned PET mask. The gel bilayer was transferred onto the SiBS/PDMS encapsulation film. The gel bilayer on the SiBS/PDMS film was covered with another PDMS/SiBS film and sealed by ironing at 140 °C.

### Test Humidity Independent Impedance of the Gel Bilayer

The encapsulated gel bilayer and the encapsulated bare ion gel single layer were placed in a chamber (TH3‐PE, Jeio Tech, Korea) at 25 °C, R.H. = 30%, or R.H. = 90% for 120 h. The electrochemical impedance spectroscopy of the gel bilayer was measured by a potentiostat (Palmsens4, Palmsens, Netherland) at AC 0.03V in the frequency range of 10^−1^–10^6 ^Hz.

### Test Humidity‐Independence and Deformation‐Independence of the Temperature Sensor

The encapsulated gel bilayer was placed in the chamber. The temperature of the chamber changed from 10 to 65 °C at R.H. = 30% and R.H. = 90%. Impedance spectroscopy of the bilayer gel was measured at every 5 °C increment. The sensor was uniaxially stretched to 50% and placed in the chamber of R.H. = 90%, then the temperature of the chamber changed from 10°C to 65°C, and impedance was measured at every 5°C increment.

### Test Sensor Performance During Water‐and‐Drying Cycles

The sensor was put in a water pot and the water temperature changed from 25 to 60 °C. After impedance measurement in water, the sensor was placed on the hot plate, and the temperature increased from 25 to 60 °C. During the changes in temperature, impedance spectroscopy of the temperature sensor was obtained. The impedance measurement in the water‐and‐hot plate was repeated to test the stability of sensor performance. The measured temperature was compared with the values measured with the IR camera (T885 V2, TESTO). The ion temperature sensor was attached to the elbow joint with a double‐sided medical tape (9834, 3M). The body temperature was measured by the ion gel sensor and the IR camera before push‐up, immediately after 100 push‐ups, and 10 m break. This study was approved by the POSTECH Ethics Committee (PIRB‐2021‐E062) and the subject's consent was obtained prior to the experiment.

### Test Long‐Term Sensor Performance

The sensor was placed in the chamber with high humidity of 90% R.H. The temperature changed from 1 to 60 °C. The next day, the sensor was put on the hot plate and measured the temperature change from 25°C to 60°C. After one day, the sensor was placed in a water bath and the temperature was measured from 25 to 60 °C. On the last day, a temperature sensor was attached to the elbow to measure body temperature before exercise, immediately after exercise, and after 10 min break. During the changes in temperature, the charge relaxation time of the ion gel temperature sensor was measured by the potentiostat. The set temperature was measured by the IR camera.

### Statistical Analysis for the Master Curve Between Temperature and Relaxation Frequency

The unstretched bilayer ion gel sensor was used to measure the temperature from 10 to 65 °C under the humid condition (R.H. = 90%, *ε* = 0%). The temperature was measured at 5 °C intervals in the sensing range. The master curve between temperature (*T*) and the relaxation frequency (ln(*τ* ^−1^)) was obtained by quadratic polynomial fitting. The number of points (n) was 12 and the *R* ^2^ value was 0.9996. We used Microsoft Excel for the calculation.

## Conflict of Interest

The authors declare no conflict of interest.

## Supporting information

Supporting Information

## Data Availability

The data that support the findings of this study are available on request from the corresponding author. The data are not publicly available due to privacy or ethical restrictions.
