# Supplementary material for: Water‐Saturated Ion Gel for Humidity‐Independent High Precision Epidermal Ionic Temperature Sensor
Source: Adv Sci (Weinh). 2022 Mar 25;9(16):2200687. doi: 10.1002/advs.202200687 (PMC9165521; doi:10.1002/advs.202200687)
Supplement: Supplementary file 1 — Supporting Information [file ADVS-9-2200687-s001.pdf]

Supporting Information

Water-Saturated Ion Gel for Humidity-Independent High Precision Epidermal Ionic Temperature Sensor

*Hyun Woo Kim, Eunseo Kim, Joosung Oh, Hyomin Lee, Unyong Jeong**

H.W. Kim, J. Oh, Prof. U. Jeong
Department of Materials Science and Engineering, Pohang University of Science and Technology (POSTECH), 77 Cheongam-Ro, Nam-Gu, Pohang, Gyeongsangbuk-Do, 37673, Republic of Korea.
Email: ujeong@postech.ac.kr

E. Kim, Prof. H. Lee
Department of Chemical Engineering, Pohang University of Science and Technology (POSTECH), 77 Cheongam-Ro, Nam-Gu, Pohang, Gyeongsangbuk-Do, 37673, Republic of Korea.

Supporting text S1. Measurement of the diffusion coefficient from the DOSY NMR

Figure S2a shows ^{1}H NMR spectra of the dry ion gel and the water-saturated ion gel. There were two peaks at δ = 2.81 ppm and 4.82 ppm in the water-saturated ion gel, but they were not observed in the NMR spectrum of the dry ion gel. The peak at δ = 2.81 ppm represents water molecules mixed with the hydrophobic gel and the peak at δ = 4.82 ppm indicates existence of phase-separated water.[1] From these two peaks and the DOSY NMR, the diffusion coefficient of water was obtained to be 2.53×10^{-4} mm^2/s in the water-saturated ion gel.

In the water-saturated hydrogel layer (**Figure S2b**), the chemical shifts at δ = 3.4, 3.5, and 3.7 ppm are from glycerol, and the peaks at δ = 1.5 and 2.1 ppm correspond to polyacrylamide.[2,3] From the water peak at δ = 4.7 ppm, the diffusion coefficient was obtained to be 1.25×10^{-3} mm^2/s from the DOSY NMR.

Supporting text S2. Schwarz-Christoffel transformation of the coplanar electrode structure

The Schwarz-Christoffel transformation is a mathematical technique allowing the analysis of potential field in a coplanar structure by a potential field in the parallel structure. Since the flux and potential between points or lines are conserved before and after conformal transformation of lines and points,[4] it has been applied to calculate physical properties such as resistance, capacitance, or electric field in complex electrode structures. [5-7]

Figure 3b exhibits the Schwarz-Christoffel transformation for the coplanar electrode system. The coplanar electrode system is depicted in the t-plane, which is the complex plane. The electrodes are on the x-axis. The upper plane of the t-plane is conformal-transformed to the rectangle on the w-plane with the Schwarz-Christoffel equation, $\frac{t}{D/2} = sn(2Kw, k)$,where sn() is the Jacobian elliptic function, k is a geometric factor which is $k = \frac{D}{D+2W}$, and K(k) is the complete elliptic integral of the first kind which is $K(k) = \int_0^1 \frac{dt}{\sqrt{(1-t^2)(1-k^2t^2)}}$. As a result, the upper half t-plane is transformed to the rectangle composed of 4 points that are (1/2, K(k`)/2K(k)), (1/2, 0), (-1/2,0), and (-1/2, K(k`)/2K(k)). And k' is the complementary geometric factor which is $k = \sqrt{1-k^2}$. Through the Schwarz-Christoffel transformation, the coplanar electrode system is transformed to the simple parallel electrode system, where the width (W) of electrode is K(k`)/2K(k) and the distance (d) between electrodes is 1. By using

the formula for R and C, $R = \rho \frac{d}{WL}$ and C$= \varepsilon \frac{WL}{d}$ in the parallel electrode structure, it is possible to calculate resistance and capacitance in the coplanar electrode structure as R $=$ $\rho \frac{2K(k)}{K(k')\,L}$ and $C = \varepsilon \frac{K(k')\,L}{2K(k)}$.

References

[1] K. Oka, T. Shibue, N. Sugimura, Y. Watabe, B. Winther-Jensen, H. Nishide, *Sci. Rep.* **2019**, *9*, 223.

[2] M. Delample, N. Villandier, J.-P. Douliez, S. Camy, J.-S. Condoret, Y. Pouilloux, J. Barrault, F. Jérôme, *Green Chem.* **2010**, *12*, 804.

[3] L. Feng, H. Zheng, B. Gao, S. Zhang, C. Zhao, Y. Zhou, B. Xu, *RSC Adv.* **2017**, *7*, 28918.

[4] K. J. Binns, P. J. Lawrenson, *Analysis and Computation of Electric and Magnetic Field Problems 2nd Edition*, Elsevier, **1973**.

[5] J. S. Wei, *IEEE J. Quantum Electron.* **1977**, *13*, 795.

[6] R. Igreja, C. J. Dias, *Sens. Actuators A: Phys.* **2004**, *112*, 291.

[7] C. E. Murray, J. M. Gambetta, D. T. McClure, M. Steffen, *IEEE Trans. Microw. Theory Tech.* **2018**, *66*, 3724.

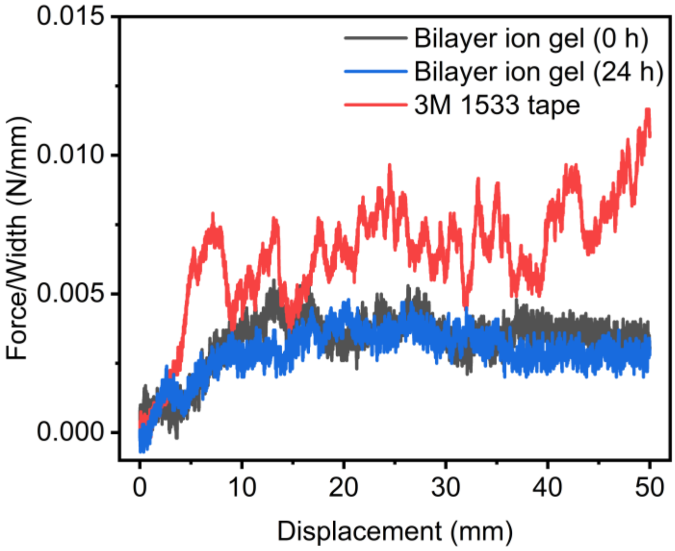

Figure S1. Comparison of the peeling tests for the gel layer interface. For the gel bilayer, the tests were performed immediately after the fabrication (black) and 24 h after fabrication (blue). The peeling test result between the commerical 3M 1533 tape and a slide glass is compared as a reference (red).

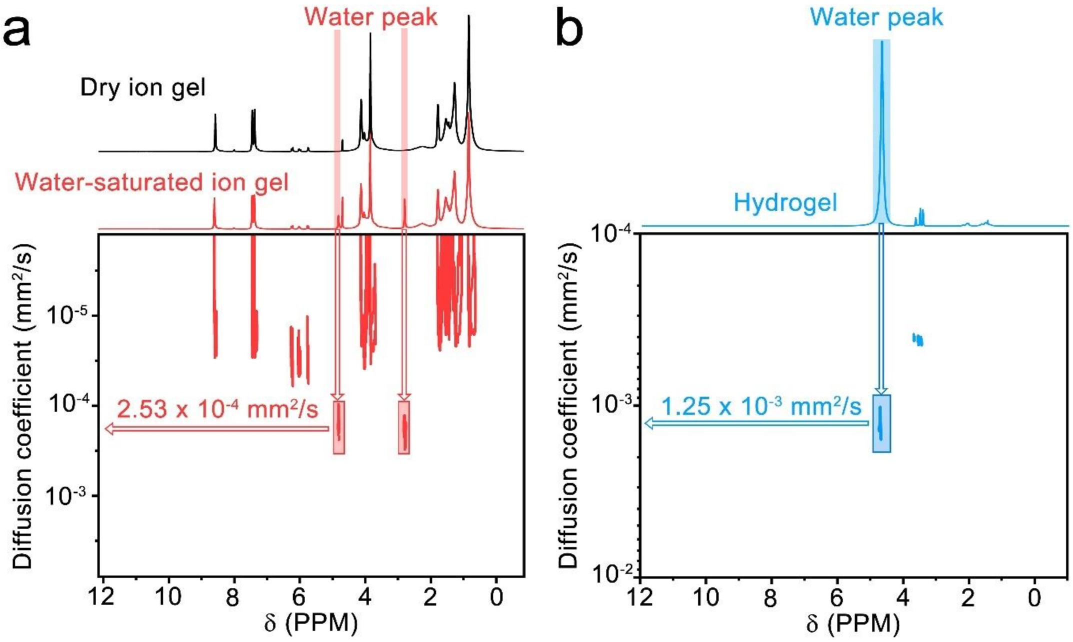

Figure S2. (a) ^{1}H NMR spectra of the dry ion gel (black) and the water-saturated ion gel (red). The NMR DOSY spectrum of the water-saturated ion gel is shown together. **(b)** ^{1}H NMR spectrum of the hydrogel (blue) and its NMR DOSY spectrum. The numbers are the diffusion coefficients of water in the water-saturated ion gel and the hydrogel.

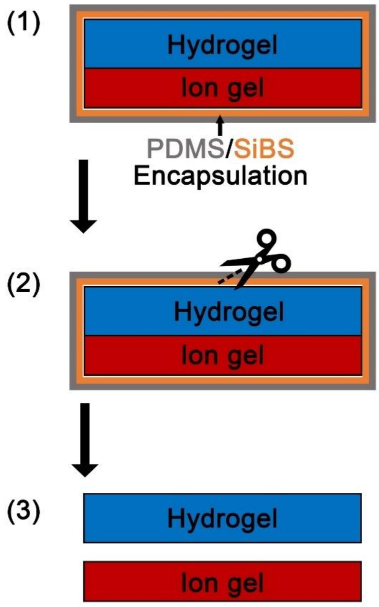

Figure S3. Scheme of measuring the water concentration in the hydrogel layer and the ion gel layer. The hydrogel layer and the ion gel layer were prepared separately and physically contacted. The PDMS/SiBS passivation layer covered the whole surfaces. The sample was tested at different conditions. Immediately after cutting the passivation layer, weight of the detached gel layers were measured. This process provided the relative concentration of water in the layers, which was the partitioning coefficient of water in the gel bilayer.

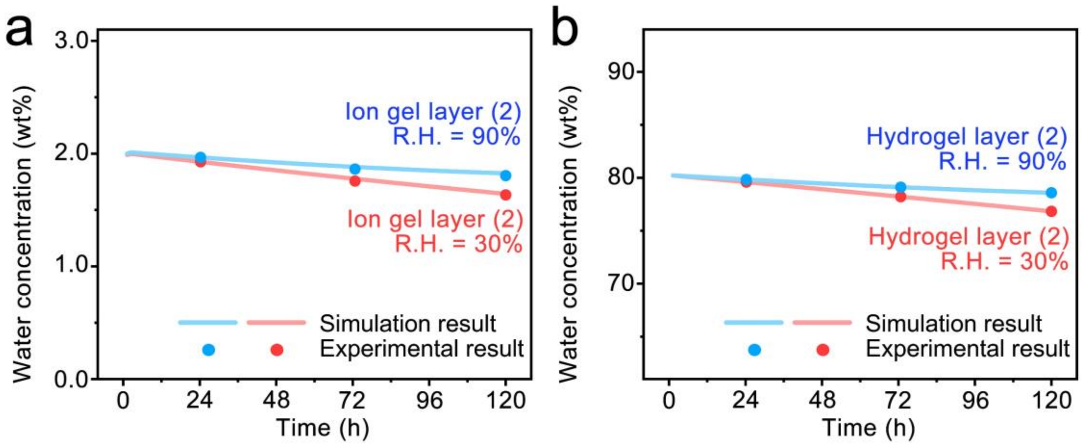

Figure S4. Computational (solid line) and experimental (symbols) results of the water concentration change in **(a)** the ion gel layer of the gel bilayer and **(b)** the hydrogel layer of the gel bilayer at R. H. = 30% (red) and 90%(blue) for 120 h.

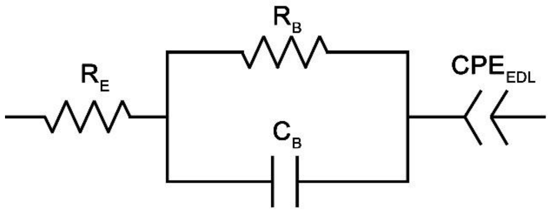

Figure S5. General equivalent circuit model of the ion gel composed of CPE by EDL (CPE_{EDL}), bulk resistance (R_B), bulk capacitance (C_B), and electrode resistance (R_E).

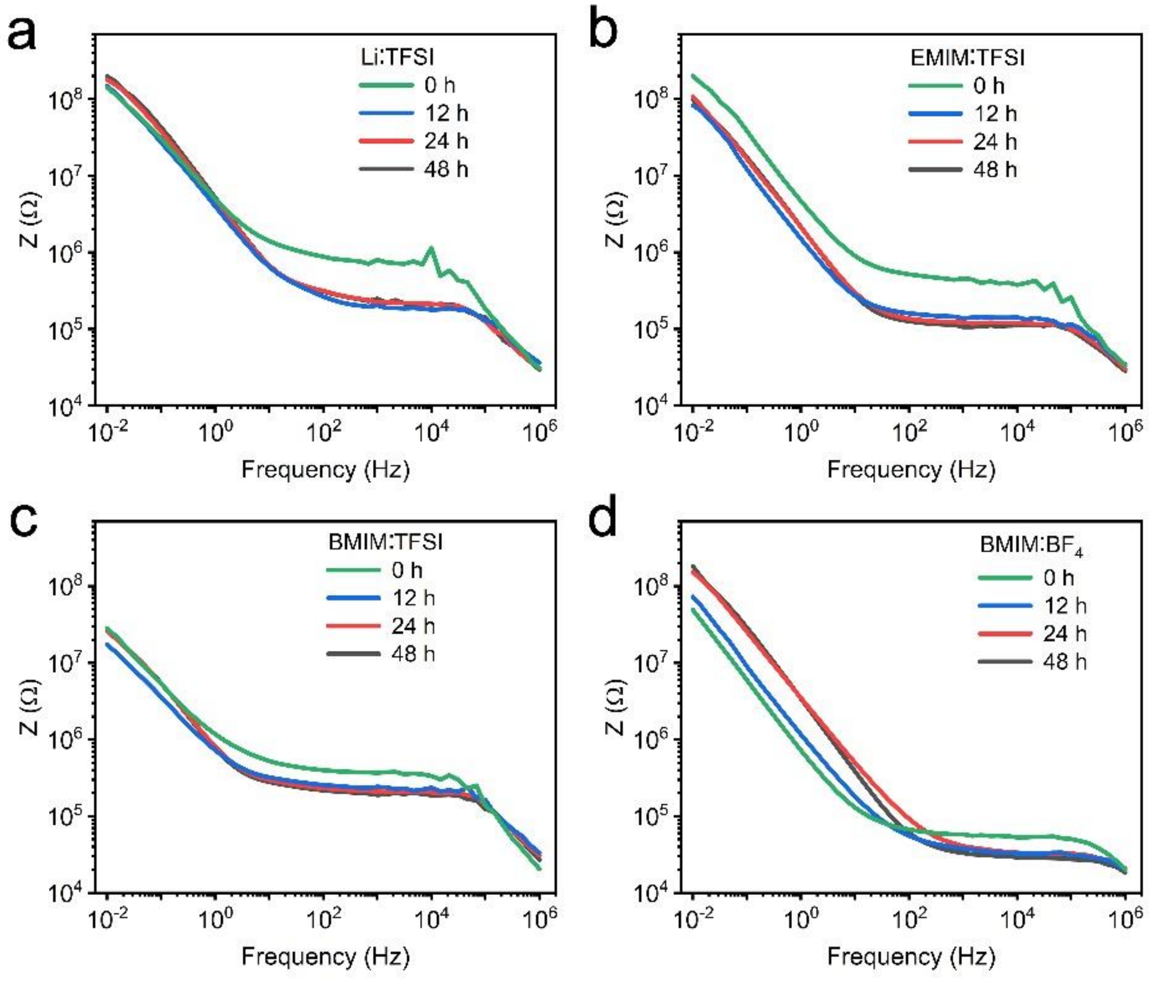

Figure S6. Bode plots of the various ion gels formed in the PEG-DA gel network containing different ionic molecules; **(a)** Li:TFSI, **(b)** EMIM:TFSI, **(c)** BMIM:TFSI, **(d)** BMIM:BF_4. All the samples were measured at 25°C and R. H. = 90% for 48 h.

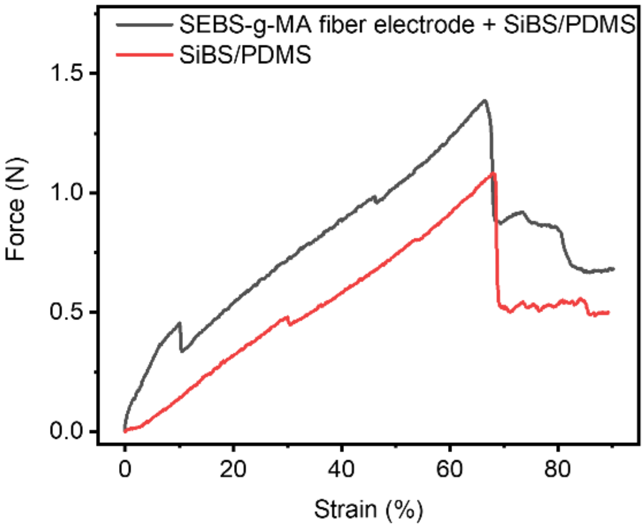

Figure S7. Strain-Force curve of the bare SiBS/PDMS film (red) and the same one atttahced with the SEBS-g-MA fiber electrode (black).

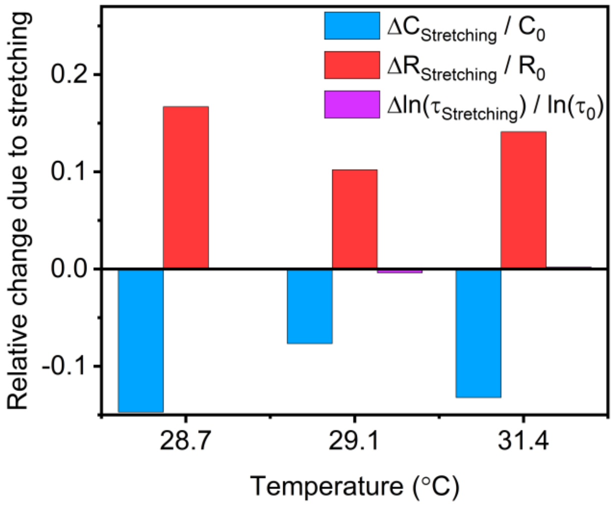

Figure S8. Relative changes of the capacitance (blue), resistance (red), and charge relaxtion time (purple) of the ion gel sensor by uniaxial stretching at 28.7°C, 29.1°C, and 31.4°C.
